# Early vascular unclamping reduces warm ischaemia time in robot-assisted laparoscopic partial nephrectomy

**DOI:** 10.12688/f1000research.6276.1

**Published:** 2015-05-06

**Authors:** Kevin Lah, Devang Desai, Charles Chabert, Christian Gericke, Troy Gianduzzo

**Affiliations:** 1 Royal Brisbane and Women's Hospital, Brisbane, Queensland, 4006, Australia; 2 University of Queensland School of Medicine, Brisbane, Queensland, 4006, Australia; 3 The Wesley Hospital, Brisbane, Queensland, 4066, Australia; 4 The Wesley Research Institute, Brisbane, Queensland, 4066, Australia

**Keywords:** Robot-assisted laparoscopy, Partial nephrectomy, Renal function, Renal Mass

## Abstract

**Introduction**: The aim of this study was to assess the outcomes of early vascular release in robot-assisted laparoscopic partial nephrectomy (RAPN) to reduce warm ischaemia time (WIT) and minimise renal dysfunction. RAPN is increasingly utilised in the management of small renal masses. To this end it is imperative that WIT is kept to a minimum to maintain renal function.

**Methods: **RAPN was performed via a four-arm robotic transperitoneal approach. The renal artery and vein were individually clamped with robotic vascular bulldog clamps to allow cold scissor excision of the tumour. The cut surface was then sutured with one or two running 3-0 V-Loc ^TM^ sutures, following which the vascular clamps were released. Specific bleeding vessels were then selectively oversewn and the collecting system repaired. Renorrhaphy was then completed using a running horizontal mattress 0-0 V-Loc ^TM^ suture.

**Results**: A total of 16 patients underwent RAPN with a median WIT of 15 minutes (range: 8-25), operative time 230 minutes (range: 180-280) and blood loss of 100 mL (range: 50-1000). There were no transfusions, secondary haemorrhages or urine leaks. There was one focal positive margin in a central 5.5 cm pT3a renal cell carcinomas (RCC). Long-term estimated glomerular filtration rate (eGFR) was not significantly different to pre-operative values.

**Conclusion**: In this patient series, early vascular release effectively minimised WIT and maintained renal function without compromising perioperative safety.

## Introduction

The incidence of diagnoses of renal masses is on the rise with increased use of abdominal imaging ^1^. Nephron-sparing surgery (NSS), such as robotic-assisted laparoscopic partial nephrectomy (RAPN) is increasingly recommended to preserve long-term renal function ^2, 3^. This is particularly important given the increasing prevalence of diabetes mellitus and hypertension in the community ^4, 5^. The American Urological Association recommends NSS as the treatment of choice for most T1 renal masses ^6^. NSS has been shown to reduce the long-term risks of renal dysfunction, and cardiovascular morbidity, as well as overall mortality when compared to radical nephrectomy ^7, 8^. The duration of warm ischaemia time (WIT) during partial nephrectomy (PN) is critically important in achieving these outcomes. Thompson and colleagues ^9^ found that a WIT of 25 minutes or longer was significantly associated with new onset of stage IV chronic kidney disease and concluded that “every minute of ischaemia counts” in the preservation of renal function ^10^. Ongoing refinement of the technique over the last decade to minimise ischaemia time has been the challenge in mastering PN.

Laparoscopic PN (LPN) is a not a procedure for any novice laparoscopic urologist ^11^. The difficulties in tumour excision and reconstruction of the collecting system and renal cortex can be improved by the use of robot-assisted laparoscopic instruments. This allows a greater degree of motion and dexterity which may allow the surgeon to reduce WIT. Other techniques to decrease WIT have included the use of intra-arterial hypothermic perfusion ^12^, intra-corporeal placement of ice slush ^13^ and specific refinements in clamping renal artery and vein, including the early vascular release technique ^14^. This study presents the initial experience of early vascular release as a means to minimise WIT during RAPN.

## Methods

With UnitingCare Health Ethics Committee approval, nr. 2013.25.96, the outcomes of RAPN of two oncology fellowship-trained surgeons (TG and CC) were analysed. Data from 16 consecutive patients was prospectively collected between July 2011 and September 2013.

Pre-operative data included: age, gender, body mass index, American Society of Anesthesiologists Physical status classification (ASA), side of the mass, pre-operative renal function (estimated glomerular filtration rate), tumour size on imaging and relevant medical history (i.e. previous abdominal surgery). Intraoperative data included: operative duration, console time, WIT, estimated blood loss and intraoperative complications. Operative duration was defined as skin-to-skin time, and console time defined as the time during which the robotic interface was used during the procedure. Perioperative data included: complications, day 1 post-operative renal function and length of hospital stay. Post-operative data included renal function at 6 months, and tumour histology, stage, margin and size. Pathological analysis was performed by a single uropathologist experienced in partial nephrectomy assessment.

RAPN was performed via a four-arm transperitoneal approach in a modified lateral position with a 30° tilt and the table at 20–30° contralateral tilt. An ipsilateral ureteric catheter is routinely used to allow retrograde instillation of methylene blue to check collecting system integrity. The kidney was mobilized in the standard fashion whereby the colon was reflected medially, the ureter elevated with the fourth arm and the duodenum kocherised for right-sided tumours. The renal vessels were then isolated and looped with vessel loops. Gerota’s fascia was then incised and the kidney defatted in order to localise the mass. Care was taken to leave perinephric fat directly on the mass. The renal mass was then assessed with intraoperative ultrasound to ascertain its depth and to plan the margin of incision. Both renal artery and vein were clamped using robotic bulldog clamps and the tumour excised with cold scissor dissection. Renorrhaphy was performed using one or two 2-0 V-Loc ^TM^ absorbable polyglyconate knotless sutures (Covidien Inc.), following which the bulldog clamps were released. This early release allowed specific bleeding vessels to be immediately positively identified and specifically suture ligated with figure-of-8, 3-0 vicryl sutures. Collecting system defects were specifically repaired and the integrity assessed with retrograde instillation of methylene blue. Cortical reconstruction was performed using a single, running horizontal mattress 12 inch 0-0 V-Loc ^TM^ suture. Floseal ^®^ (Baxter Corp.) was applied to the closed defect.

### Statistical data analysis

Numerical data was summarised using median and range (Microsoft Excel), and analysed using the Wilcoxon signed-rank test ( www.socscistatistics.com) where appropriate. A P < 0.05 was considered to indicate statistical significance. Demographics and categorical data were summarised in table format.

## Results

A total of 16 patients underwent RAPN. There were ten males and six females with a median age of 66.5 (range 48 to 80 years). There were nine left sided lesions and seven right sided lesions. The majority of the masses were exophytic with a median size of 2.65 cm. Table 1 shows the demographics.

**Table 1.** Demographics.

N	16
Gender (M:F)	10:6
Age Median (Range)	66.5 years (48 – 80)
ASA	2
BMI Median (Range)	27.1 (20.1 – 35.3)
Tumour side (L:R)	9:7
Exophytic : Endophytic tumour	13:3

ASA: American Society of Anesthesiologists Physical status classification

Perioperative data are recorded in Table 2. Day 1 eGFR was marginally reduced compared to pre-operative levels (p < 0.01). However this was not clinically significant and by 6 months eGFR had returned to baseline (p = 0.11). The median operative time was 230 minutes with 192.5 minutes of console time. Median WIT was 15 minutes and median blood loss was 100 mL. The median hospital length of stay was 2 days. There were no transfusions, urine leaks, or post-operative haemorrhage. Histopathology demonstrated nine clear cell renal cell carcinomas (RCC), three papillary RCC, three angiomyolipoma and three eosinophilic variant clear cell RCC ( Table 3). Tumour abutted the resection margin in one case of a central 5.5 cm mass which demonstrated renal sinus and vascular invasion on frozen section. A completion nephrectomy was then immediately performed given these high-risk features and also given that the contra-lateral kidney and pre-operative renal function were normal. There was no residual tumour in the remaining kidney. One patient had a grade 1 Clavien-Dindo classification who had self-resolved neuralgic pain. No cases were converted to an open operation. Our study is compared with international data in Table 4.

**Table 2.** Perioperative data.

Pre-op eGFR Median (Range)	74 ml/min/1.73 m ^2^ (53–90)
Day 1 Post-op eGFR Median (Range)	63 ml/min/1.73 m ^2^ (39–90) [p < 0.01]
6 Month eGFR Median (Range)	70 mL/min/1.73 m ^2^ (53–90) [p = 0.11]
Operative time (Range)	230 mins (180–280)
Console time (Range)	192.5 mins (150–230)
WIT (Range)	15 mins (8–25)
Blood loss Median (Range)	100 ml (50–1000)
Length of stay	2 days (1–6)
Complications	Neuralgic pain self resolved

eGFR: estimated glomerular filtration rate. [p value] compared with pre-op.

**Table 3.** Histopathology.

Clear Cell RCC	9
Papillary RCC	3
AML	1
Eosinophilic variant clear cell RCC	3
Positive Margins	1

RCC: Renal cell carcinoma; AML: Angiomyolipoma

**Table 4.** Study comparison.

Median (Range)	Current Study (n=16)	San Francisco *et al.* (n=12)
Operative time	230 mins (180–280)	227 mins (176–315)
WIT	15 mins (8–25)	16 mins (11–25)
Blood loss	100 mL (50–1000)	150 mL (50–500)
Length of stay	2 days (2–6)	2 days (2–7)
Positive margins	1 (6.25%)	1 (8%)

WIT: Warm ischaemia time

Raw data Pre-, post- and perioperative data for patients undergoing robot-assisted laparoscopic partial nephrectomy ^23^.

## Discussion

RAPN is an effective surgical alternative in NSS in which the ultimate goal is to achieve the “trifecta” of a negative cancer margin, minimal decrease in renal function and an absence of complications ^14^. The use of robotic technology can assist in achieving these outcomes and in particular, minimise renal dysfunction by reducing WIT. Laparoscopic partial nephrectomy not only has a steep learning curve to achieve acceptable WIT but also requires skills that are challenged by its technical difficulties, including the use of instruments that have limited degrees-of-freedom. The robot application in PN allows a three-dimensional vision with magnification and instrument arms that are versatile with its EndoWrist ^®^ technology, providing increased angle and maneuverability for tumour excision and repair of the renal defect ^15^.

Various methods have been employed in the past to lessen the ischaemic injury. Intra-arterial hypothermic perfusion was used in early series of LPN ^12^ as was intra-corporeal placement of ice slush ^13^. However endovascular hypothermia came with the added risks of placing an arterial catheter and administration of extra fluid for patients with poor cardiopulmonary performance status. Intra-corporeal cooling reduced the working space and exposure of the hilum.

There has been an ongoing refinement of clamping techniques to reduce WIT, progressing from conventional clamping of both renal artery and vein ^10^ to early unclamping ^14, 16, 17^. With the introduction of early unclamping, where the clamp is released once a central medullary running suture is placed and the rest of the kidney repaired with the revascularised perfused kidney, Nguyen and Gill ^16^ were able to decrease WIT in LPN from 31.3 minutes to 13.9 minutes. The overall complications including estimated blood loss were not significantly different compared with the standard clamping technique. Furthermore, surgeons have pushed the boundaries of minimal ischaemia by selectively micro-clamping arteries supplying the tumour ^18, 19^ and in one study, without clamping and aiming for “zero ischaemia” ^20^. This clamp-less group of eight patients had significantly reduced operative time but an increased blood loss. The transfusion and renal dysfunction were similar to the clamped group.

The technique of early vascular release has been translated from LPN to RAPN ^17^. San Francisco *et al.* described 12 patients who underwent RAPN with early vascular release ( Table 4) ^17^. In that series, the median WIT was 16 minutes, median operative time 227 minutes, median estimated blood loss 150 mL and length of stay 2 days. These results are comparable to the present study ( Table 4).

Kaouk and colleagues ^21^ reported their single-institution’s 252 RAPNs with early unclamping technique when deemed appropriate. Their study confirmed that the longer the duration of WIT, the greater the decrease in renal dysfunction at 1 month. Kucharczyk and colleagues reported an Australian series of 50 consecutive patients undergoing RAPN by a single surgeon ^22^. The mean WIT was 17.8 minutes, operative time was 151 minutes and estimated blood loss was 171.1 ml. They achieved no positive malignant surgical margins and a clinically stable renal function post-operatively.

In this series, the outcomes of early vascular release were comparable to the literature with short WIT and no morbidity from intraoperative or postoperative haemorrhage or urine leak. The limitations of this study include a small sample size, and the lack of a randomised comparison to other techniques.

In conclusion, early vascular release following tumour excision during RAPN resulted in short WIT with minimal morbidity and preserved renal function.

## Data availability

*F1000Research*: Dataset 1. Raw data, 10.5256/f1000research.6276.d46769 ^23^

## Consent

All patients gave consent for collection of data for research purposes.
