# Supplementary material for: Early vascular unclamping reduces warm ischaemia time in robot-assisted laparoscopic partial nephrectomy
Source: F1000Res. 2015 May 6;4:108. [Version 1] doi: 10.12688/f1000research.6276.1 (PMC4431378; doi:10.12688/f1000research.6276.1)
Supplement: Peer review history

# Peer review: Referee response for version 1

**DOI:** 10.5256/f1000research.6732.r8577

**Published:** 2015-05-13

**Authors:** Mark Louie-Johnsun

**Affiliations:** 1 Department of Urology, Wyong Hospital, Sydney, NSW, Australia

This article highlights the early solid results of two oncology fellowship-trained surgeons performing robot assisted laparoscopic partial nephrectomy. The short WIT with minimal perioperative complications highlights this complex operation can be performed expertly in well trained surgeons even in their early experience.

I have read this submission. I believe that I have an appropriate level of expertise to confirm that it is of an acceptable scientific standard.

# Peer review: Referee response for version 1

**DOI:** 10.5256/f1000research.6732.r8579

**Published:** 2015-05-12

**Authors:** Prem Rashid

**Affiliations:** 1 Department of Urology, Port Macquarie Base Hospital, Rural Clinical School, The University of New South Wales, Sydney, NSW, Australia

This paper on the early experience of robotic LPN highlights the complexity of the procedure and the possible outcomes with good background preparation. The results are helpful to guide those embarking on LPN or robotic assisted LPN. It is well written. The methodology and conclusions are sound.

I have read this submission. I believe that I have an appropriate level of expertise to confirm that it is of an acceptable scientific standard.
